# Impact of Portable Normothermic Blood-Based Machine Perfusion on Outcomes of Liver Transplant: The OCS Liver PROTECT Randomized Clinical Trial

**DOI:** 10.1001/jamasurg.2021.6781

**Published:** 2022-01-05

**Authors:** James F. Markmann, Marwan S. Abouljoud, R. Mark Ghobrial, Chandra S. Bhati, Shawn J. Pelletier, Amy D. Lu, Shane Ottmann, Tarunjeet Klair, Corey Eymard, Garrett R. Roll, Joseph Magliocca, Timothy L. Pruett, Jorge Reyes, Sylvester M. Black, Christopher L. Marsh, Gabriel Schnickel, Milan Kinkhabwala, Sander S. Florman, Shaheed Merani, Anthony J. Demetris, Shoko Kimura, Michael Rizzari, Ashish Saharia, Marlon Levy, Avinash Agarwal, Francisco G. Cigarroa, James D. Eason, Shareef Syed, W. Kenneth Washburn, Justin Parekh, Jang Moon, Alexander Maskin, Heidi Yeh, Parsia A. Vagefi, Malcolm P. MacConmara

**Affiliations:** 1 Massachusetts General Hospital, Boston; 2 Henry Ford Transplant Institute, Detroit, Michigan; 3 Houston Methodist, Houston, Texas; 4 Virginia Commonwealth University, Richmond; 5 University of Virginia, Charlottesville; 6 Tampa General, Tampa, Florida; 7 Johns Hopkins, Baltimore, Maryland; 8 UT Health Science Center, San Antonio, Texas; 9 University of Tennessee Health Science Center, Memphis; 10 UCSF, San Francisco, California; 11 Emory University Hospital, Atlanta, Georgia; 12 University of Minnesota, Minneapolis; 13 University of Washington, Seattle; 14 The Ohio State University, Columbus; 15 Scripps Clinic and Scripps Green Hospital, San Diego, California; 16 University of California, San Diego, La Jolla; 17 Montefiore Medical Center, Bronx, New York; 18 Mount Sinai Health System, New York, New York; 19 University of Nebraska Medical Center, Omaha; 20 University of Pittsburgh Medical Center, Pittsburgh, Pennsylvania; 21 University of Texas Southwestern Medical Center, Dallas

## Abstract

**Importance:**

Ischemic cold storage (ICS) of livers for transplant is associated with serious posttransplant complications and underuse of liver allografts.

**Objective:**

To determine whether portable normothermic machine perfusion preservation of livers obtained from deceased donors using the Organ Care System (OCS) Liver ameliorates early allograft dysfunction (EAD) and ischemic biliary complications (IBCs).

**Design, Setting, and Participants:**

This multicenter randomized clinical trial (International Randomized Trial to Evaluate the Effectiveness of the Portable Organ Care System Liver for Preserving and Assessing Donor Livers for Transplantation) was conducted between November 2016 and October 2019 at 20 US liver transplant programs. The trial compared outcomes for 300 recipients of livers preserved using either OCS (n = 153) or ICS (n = 147). Participants were actively listed for liver transplant on the United Network of Organ Sharing national waiting list.

**Interventions:**

Transplants were performed for recipients randomly assigned to receive donor livers preserved by either conventional ICS or the OCS Liver initiated at the donor hospital.

**Main Outcomes and Measures:**

The primary effectiveness end point was incidence of EAD. Secondary end points included OCS Liver ex vivo assessment capability of donor allografts, extent of reperfusion syndrome, incidence of IBC at 6 and 12 months, and overall recipient survival after transplant. The primary safety end point was the number of liver graft–related severe adverse events within 30 days after transplant.

**Results:**

Of 293 patients in the per-protocol population, the primary analysis population for effectiveness, 151 were in the OCS Liver group (mean [SD] age, 57.1 [10.3] years; 102 [67%] men), and 142 were in the ICS group (mean SD age, 58.6 [10.0] years; 100 [68%] men). The primary effectiveness end point was met by a significant decrease in EAD (27 of 150 [18%] vs 44 of 141 [31%]; *P* = .01). The OCS Liver preserved livers had significant reduction in histopathologic evidence of ischemia-reperfusion injury after reperfusion (eg, less moderate to severe lobular inflammation: 9 of 150 [6%] for OCS Liver vs 18 of 141 [13%] for ICS; *P* = .004). The OCS Liver resulted in significantly higher use of livers from donors after cardiac death (28 of 55 [51%] for the OCS Liver vs 13 of 51 [26%] for ICS; *P* = .007). The OCS Liver was also associated with significant reduction in incidence of IBC 6 months (1.3% vs 8.5%; *P* = .02) and 12 months (2.6% vs 9.9%; *P* = .02) after transplant.

**Conclusions and Relevance:**

This multicenter randomized clinical trial provides the first indication, to our knowledge, that normothermic machine perfusion preservation of deceased donor livers reduces both posttransplant EAD and IBC. Use of the OCS Liver also resulted in increased use of livers from donors after cardiac death. Together these findings indicate that OCS Liver preservation is associated with superior posttransplant outcomes and increased donor liver use.

**Trial Registration:**

ClinicalTrials.gov Identifier: NCT02522871

## Key Points

**Question:**

Can oxygenated portable normothermic perfusion of deceased donor livers for transplant improve outcomes compared with the current standard of care using ischemic cold storage?

**Findings:**

In this multicenter randomized clinical trial of 300 recipients of liver transplants with the donor liver preserved by either normothermic perfusion or conventional ischemic cold storage, normothermic machine perfusion resulted in decreased early liver graft injury and ischemic biliary complications and greater organ utilization.

**Meaning:**

In this study, portable normothermic oxygenated machine perfusion of donor liver grafts resulted in improved outcomes after liver transplant and in more livers being transplanted.

## Teaser

This multicenter randomized clinical trial compares the use of oxygenated portable normothermic machine perfusion of deceased donor livers for transplant vs conventional ischemic cold storage to assess liver function preservation and outcomes among recipients of a liver allograft.

## Introduction

Liver transplant provides lifesaving treatment for patients with end-stage liver disease. An inadequate supply of suitable donor organs has resulted in prolonged waiting times and high waiting list mortality rates.^1,2^ Historically, liver preservation has relied almost exclusively on ischemic cold storage (ICS).^3,4^ However, ICS subjects liver allografts to ischemic injury and progressive organ deterioration, even at 4 °C.^5^ In addition, ICS precludes ex vivo optimization or assessment of liver function, leaving the cumulative effects of donor quality, preservation and reperfusion injury to be revealed only after implantation.^6,7,8^ The inherent uncertainties of livers preserved by ICS foster conservatism by transplant clinicians when considering donor organ quality, ultimately leading to underuse of available donor livers for transplant. The organs from donors with advanced age, multiple comorbidities, or donation after circulatory death (DCD) status are frequently declined for transplant owing to the clinician’s concern for increased risk of early allograft dysfunction (EAD), primary nonfunction, or serious chronic complications, such as ischemic biliary complications (IBCs).^9,10^

Historically, advances in organ preservation have been linked to improved posttransplant outcomes and improved donor organ use. The concept of normothermic blood-based liver perfusion was developed to overcome the limitations of ICS.^11,12,13,14,15,16^

The multicenter International Randomized Trial to Evaluate the Effectiveness of the Portable Organ Care System (OCS) Liver for Preserving and Assessing Donor Livers for Transplantation (the PROTECT trial) is the first US randomized clinical trial for liver perfusion and was designed to overcome the limitations of ICS, limiting the period of ischemia and providing physiologic assessment of liver graft function. The PROTECT trial prespecified donor characteristics from donation after brainstem death (DBD) and DCD donors that are known to be more vulnerable to ICS associated damage.

## Methods

### Study Design

The PROTECT trial, a pivotal multicenter randomized clinical trial, compared posttransplant outcomes for recipients who received donor livers preserved using ICS or the OCS Liver (trial protocol in Supplement 1). Following initial acceptance of a donor liver for transplant, recipients were randomized 1:1 to the OCS or ICS control group through the Interactive Web Response System. The donor liver was procured at the donor site and was preserved according to randomization. All livers in the OCS Liver group were initiated on the OCS Liver device at the donor hospital. Screen failures due to the identification of intraoperative exclusion criteria in otherwise clinically suitable livers resulted in the recipient undergoing a transplant outside of the trial using a liver processed through the standard of care, ICS. When donor livers were deemed unsuitable for transplant during intraoperative physical assessment, recipients remained eligible but were treated as a new patient and rerandomized to a group at their subsequent donor offer acceptance. This practice avoided potential bias of knowing the randomization assignment on the clinical decision of donor organ acceptance (eFigure 1 in Supplement 2). The trial targeted major academic liver transplant centers and was conducted in accordance with the Humanitarian Good Clinical Practice Guidelines, with each site obtaining the appropriate institutional review board approval. This trial also followed the Consolidated Standards of Reporting Trials (CONSORT) reporting guideline. All participants gave written informed consent that was obtained in a manner consistent with the Common Rule requirements. No one received compensation or was offered any incentive for participating in this study.

### Participants

The PROTECT trial objective was to compare the safety and the effectiveness of the OCS Liver vs ICS for donors with at least 1 of the following characteristics: (1) 40 years of age or older; (2) expected total cross-clamp/cold ischemic time of 6 or more hours; (3) DCD donors if 55 years or younger; or (4) macrosteatotic livers (≤40%). Donor liver exclusion criteria included living donors, split livers, livers requiring accessory vessel reconstruction, or moderate to severe traumatic liver injury. Recipient exclusion criteria included younger than 18 years, acute or fulminant liver failure, prior solid organ or bone marrow transplant, chronic kidney failure, multiorgan transplant, ventilator dependence, or hemodynamic compromise.

### The OCS Liver

**Figure 1.**
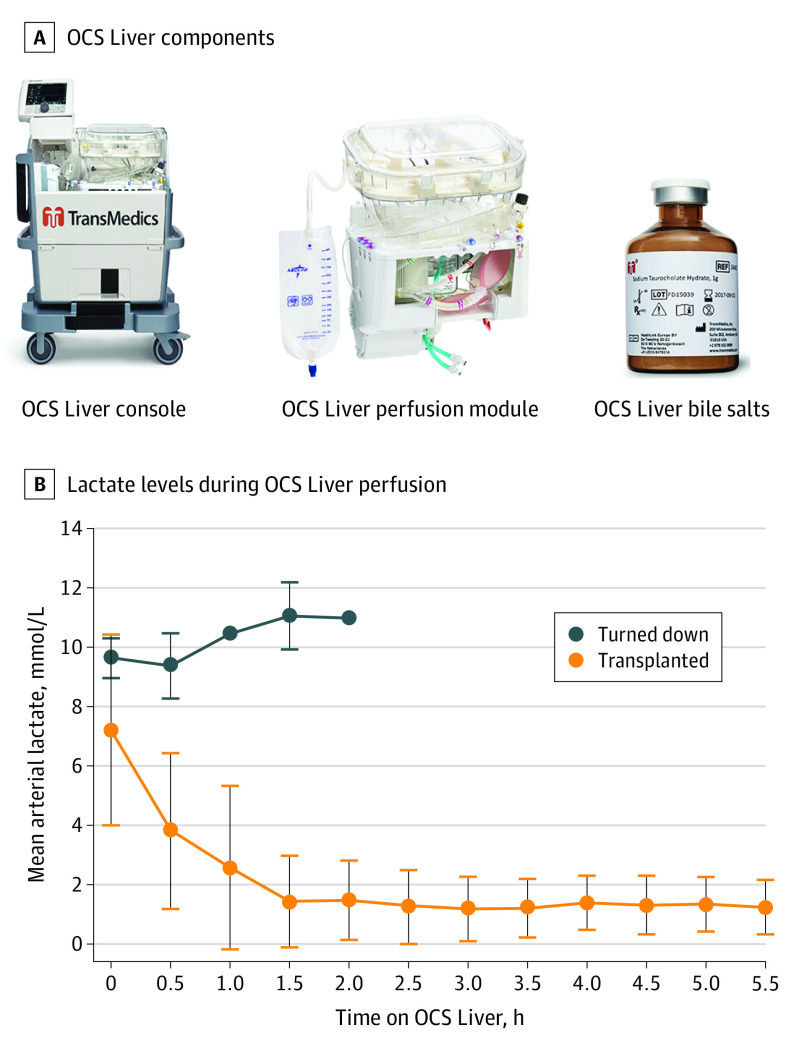
Organ Care System (OCS) Liver Components A, The OCS Liver is primed with buffered electrolyte solution, albumin, 4 to 5 units of packed red blood cells, and broad-spectrum antibiotics. Perfusion gradually increased to 2.0 to 2.5 L total flow, and temperature was set at 34 °C. The perfusate was supplemented with continuous infusion of a nutrient solution of 4% amino acids and 10% dextrose, supplemented with insulin and multivitamins. Liver perfusion hemodynamic parameters were monitored continuously and recorded throughout preservation. Serial blood gases and lactate levels were measured throughout preservation, and changes in lactate level were used to measure adequacy of perfusion. B, Donor livers that were transplanted vs clinically turned down based on OCS Liver assessment of lactate levels. Error bars represent SD.

The OCS Liver consisted of an integrated system of 3 components: the OCS Liver console, OCS Liver perfusion set, and OCS bile salt solution for infusion (Figure 1A). The OCS Liver maintains the donor liver in a nonischemic, metabolically active state by perfusing both portal venous and hepatic arterial circulations with a warm, oxygenated and nutrient-enriched, blood-based perfusate. The OCS Liver is capable of delivering high-pressure pulsatile perfusion to the hepatic arterial circulation while simultaneously delivering low-pressure, high-flow perfusion to the portal circulation with a single perfusion pump, giving the user full control over the perfusion parameters (eFigure 2 in Supplement 2).

### Procedures

After organ acceptance, potential liver recipients who had provided informed consent for participating in the PROTECT trial were randomized to OCS Liver or to ICS preservation of the donor liver before the recovery team departed for organ retrieval. During organ recovery, once donor livers were examined and accepted for transplant and for inclusion in the PROTECT trial, preservation followed the randomization assignment. For the OCS Liver instrumentation, the hepatic artery and portal vein were cannulated for inflow, and the suprahepatic cava for outflow; the common bile duct was cannulated for bile collection and quantitation. Type-specific or O Rh-negative blood was sourced from banked irradiated, leukocyte-depleted packed red blood cells.

Formalin-fixed biopsy material was received by the central laboratory, paraffin embedded, and sectioned at 4 µm prior to routine staining with both hematoxylin-eosin and CD31 (mouse monoclonal, M0823; Dako) immunostaining protocols. Sections were assessed for sectioning and staining quality before being converted to high-resolution (40×; 0.116 μm/pixel) whole-slide imagery using a Zeiss AxioScan.Z1 automated scanning system. Resultant whole-scan images were quality controlled for focus and scanning artifacts and entered into a 21 CFR Part 11 compliant imaging database for prospective scoring (treatment group and donor organ subtype were not available to the reviewing pathologist). In total, 42 histopathologic criteria were electronically recorded in the database, which included comprehensive semiquantitative assessment of severity and composition of portal, lobular, and any other notable features. We used CD31 staining to assess the integrity of the endothelium lining. At the completion of the study, all histopathologic scoring was extracted and analyzed using R, version 3.6.3 (R Foundation for Statistical Computing) for graphical and statistical data review, summary, and presentation.

### Outcomes

The primary effectiveness end point was the incidence of EAD,^10^ defined as the presence of 1 or more of the following: aspartate aminotransferase level higher than 2000 IU/L (to convert to microkatals per liter, multiply by 0.0167) within the first 7 postoperative days; bilirubin 10 mg/dL or higher (to convert to micromoles per liter, multiply by 17.104) on postoperative day 7; international normalized ratio of 1.6 or higher on postoperative day 7; or graft primary nonfunction within the first 7 days, defined as irreversible graft dysfunction leading to recipient death or emergency retransplant, in the absence of immunologic or surgical causes.

The secondary effectiveness and other critical clinical end points included the ability of the OCS Liver to monitor donor liver function throughout preservation; patient survival at day 30 or at initial hospital discharge if longer than 30 days; incidence of IBC defined as nonanastomotic ischemic strictures or bile leaks, confirmed with an endoscopic retrograde cholangiopancreatography or magnetic resonance cholangiopancreatography radiologic examination and blindly adjudicated by a 3-member independent clinical events committee; extent of reperfusion syndrome after transplant assessed by recipient’s lactate levels approximately 120 minutes after reperfusion in the recipient; and histologic assessment of the donor liver after transplant compared with baseline histologic specimen obtained before retrieval from the donor. All histopathologic evaluations were assessed using a blinded, independent core laboratory with extensive experience in liver transplant pathology.

The primary safety end point was the mean number of liver graft–related severe adverse events (LGRSAEs) per patient within the initial 30 days after liver transplant. The LGRSAEs were predefined as a primary nonfunctioning graft, IBCs, hepatic vascular complications, or liver graft infection.

### Statistical Analysis

The primary analysis population for effectiveness was the per-protocol population, consisting of all randomized recipients who received a donor liver that underwent the complete preservation procedure as per randomization assignment and had no major protocol violations. Safety was analyzed based on the as-treated population, consisting of all patients with a liver allograft. The modified intention-to-treat population consisted of all randomized patients who underwent a transplant in the PROTECT trial.

The PROTECT trial was designed to test for both noninferiority and superiority if noninferiority was met. The primary effectiveness end point was analyzed by calculating the sample proportion of patients meeting the primary effectiveness end point, as well as an exact (Clopper-Pearson) 95% CI for the corresponding population proportion. The 95% upper bound of the exact unconditional 1-sided CI based on the Farrington-Manning score was calculated for the difference between the 2 population proportions (OCS − ICS). An upper confidence limit lower than δ = 0.075 would result in rejection of the null hypothesis in favor of the alternative hypothesis and the demonstration of noninferiority of OCS to ICS for the primary effectiveness end point. In the event noninferiority was demonstrated, the Fisher exact test (2-sided) was used to assess for superiority. The secondary end points were analyzed in a manner analogous to the primary effectiveness end point.

The safety end point was analyzed by treatment group using descriptive statistics. For the mean number of LGRSAEs, the hypothesis was that the OCS Liver treatment was noninferior to the standard of care treatment, with a noninferiority margin of 1.00. The safety end point was analyzed using a 1-sided, 2-sample *t* test with an α level of .05. Statistical analyses were conducted using SAS Studio, version 9.4 (SAS Institute Inc).

## Results

**Figure 2.**
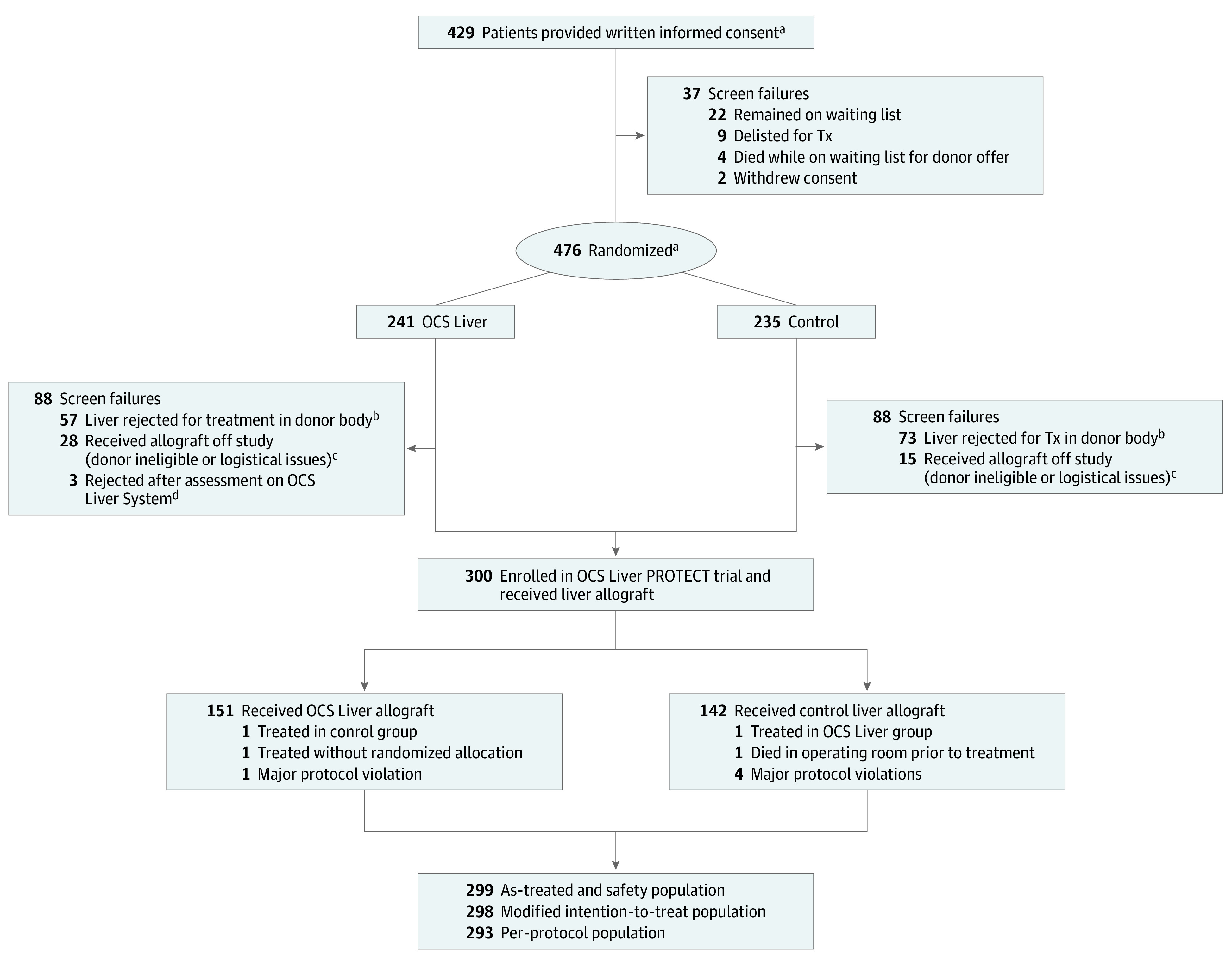
PROTECT Trial Consolidated Standards of Reporting Trials Diagram ICS indicates ischemic cold storage; OCS, Organ Care System; Tx, transplant. ^a^Some patients had more than 1 randomization event when the prior donor liver was unsuitable for clinical use after assessment in the body and the subsequent donor liver offer met study criteria. Rerandomization was performed as detailed in eFigure 1 in Supplement 2. This rerandomization process resulted in 476 randomizations in 392 patients. ^b^Of 130 livers (57 in the OCS Liver group and 73 in the ICS group) rejected for transplant in donor body after randomization, 42 (18 in the OCS Liver group and 24 in the ICS group) were rejected because the donor (after cardiac death) did not expire within 30 minutes; 31 (9 in the OCS Liver group and 22 in the ICS group) owing to clinical judgment at retrieval; 27 (13 in the OCS Liver group and 14 in the ICS group) owing to steatosis; 9 (3 in the OCS Liver group and 6 in the ICS group) showed cirrhosis or fibrosis; 4 (2 in the OCS Liver group and 2 in the ICS group) showed vasculature abnormalities or disease; 3 (3 in the OCS Liver group and 0 in the ICS group) owing to donor-recipient organ size mismatch; 2 (2 in the OCS Liver group and 0 in the ICS group) revealed liver or kidney malignant neoplasm during retrieval; and 12 (7 in the OCS Liver group and 5 in the ICS group) owing to reallocation, donor did not progress, or logistical reasons. ^c^Of 43 recipients (28 in the OCS Liver group and 15 in the ICS group) treated off study after randomization using cold storage, 39 (24 in the OCS Liver group and 15 in the ICS group) were because the donor liver did not meet eligibility owing to accessory vessels, liver hematoma or required surgical vascular repair; and 4 (4 in the OCS Liver group and 0 in the ICS group) were because of logistic reasons, including donor family did not consent to research (Organ Procurement Organization requirement), preretrieval liver biopsy could not be obtained; Organ Procurement Organization delayed operating room time, resulting in trained trial retrieval team being off call; and recipient deterioration with renal insufficiency on day of transplant. ^d^Of 3 livers from donors after cardiac death that were rejected for use after OCS Liver assessment, 2 were rejected because of increasing lactate levels despite maximizing OCS Liver perfusion parameters; and 1 because donor liver preretrieval biopsy revealed extensive bridging fibrosis.

Between November 2016 and October 2019, a total of 429 patients provided informed consent to participate in the PROTECT trial, and 300 patients were randomized and received a liver allograft (153 in the OCS Liver group and 147 in the ICS group) (Figure 2). One recipient who was to receive an ICS-perfused liver died in the operating room during surgery prior to initiating organ implantation. There were 2 treatment crossovers owing to reallocation after randomization, 1 in the OCS Liver group and 1 in the ICS group. The as-treated population consisted of 299 patients (153 in the OCS Liver group and 146 in the ICS group); 1 patient in the OCS Liver group received an allograft without having been randomized, leaving the modified intention-to-treat population consisting of 298 patients (152 in the OCS Liver group and 146 in the ICS group). After we removed the major protocol violations, the per-protocol population consisted of 293 patients (151 in the OCS Liver group and 142 in the ICS group). The donor demographic characteristics and risk factors were comparable between the 2 groups except for more DCD donors (28 of 151; 19%) in the OCS Liver group (11 of 143; 8%). The mean SD age of donors was 45.9 (14.9) years in the OCS Liver group and 46.9 (15.3) years in the ICS group. Recipient demographic characteristics and risk factors were also equivalent between the 2 groups (Table). For the OCS Liver group, the mean (SD) age was 57.2 (10.3) years, 100 recipients (66%) were men, and 51 recipients (34%) were women. For the ICS group, the mean (SD) age was 58.4 (10.1) years, 98 recipients (69%) were men, and 44 recipients (31%) were women.

**Table.** Donor and Recipient Demographic Characteristics and Risk Factors for Transplanted Organs and Recipients

Characteristic or risk factor	Donors or recipients in primary analysis per-protocol population, No. (%)		Donors or recipients in as-treated population, No. (%)	
	OCS Liver	ICS	OCS Liver	ICS
**Donor**				
No.	151	142	153	146
Age, y				
Mean (SD)	45.9 (14.9)	46.9 (15.3)	45.8 (14.9)	47.0 (15.2)
Median (range)	47.5 (10.9-83.7)	45.8 (13.0-80.6)	47.3 (10.9-83.7)	46.4 (13.0-80.6)
Age ≥40 y	102 (68)	91 (64)	102 (67)	93 (64)
Total cross-clamp time ≥6 h	47 (31)	54 (38)	48 (32)	56 (38)
DCD age ≤55 y	28 (19)	11 (8)	28 (18)	13 (9)
Steatotic liver >0% and ≤40% macrosteatosis	94 (62)	86 (61)	95 (63)	86 (59)
Multiple donor characteristics	94 (62%)	85 (58)		
**Recipient**				
No.	151	142	152	146
Age, y				
Mean (SD)	57.2 (10.3)	58.4 (10.1)	57.1 (10.3)	58.6 (10.0)
Median (range)	59.2 (19.5-76.6)	61.4 (20.8-77.8)	59.2 (19.5-76.6)	58.2 (20.8-77.8)
Sex				
Male	100 (66)	98 (69)	102 (67)	100 (68)
Female	51 (37)	44 (31)	51 (33)	46 (32)
BMI, mean (SD) [range]	29.7 (5.4) [16.3-45.5]	29.5 (5.5) [17.1-44.7]	29.7 (5.4) [16.3-45.5]	29.5 (5.5) [17.1-44.7]
MELD score				
Mean (SD)	28.4 (6.9)	28.0 (5.7)	28.4 (6.9)	28.0 (5.7)
Median (range)	29.0 (6.0-49.0)	29.0 (9.0-46.0)	29.0 (6.0-49.0)	29.0 (9.0-46.0)
Primary diagnosis				
Cholestatic diseases	9 (6)	8 (6)	9 (6)	8 (5)
Chronic hepatitis	26 (17)	35 (25)	27 (18)	36 (25)
Alcoholic cirrhosis	53 (35)	47 (33)	54 (35)	48 (33)
Metabolic diseases	6 (4)	6 (4)	6 (4)	6 (4)
Primary hepatic tumors	14 (9)	15 (11)	14 (9)	15 (10)
NASH	24 (16)	18 (13)	24 (16)	20 (14)
Other	19 (13)	13 (9)	19 (12)	13 (9)

Abbreviations: BMI, body mass index (calculated as weight in kilograms divided by height in meters squared); DCD, donor after cardiac death; ICS, ischemic cold storage; MELD, Model for End-stage Liver Disease; NASH, nonalcoholic steatohepatitis; OCS, Organ Care System.

### OCS Donor Liver Preservation Parameters, OCS Liver Use, and Critical Transplant Times

Donor livers were perfused with the OCS Liver for a mean (SD) of 276.6 (117.4) minutes and were maintained in near physiologic condition as shown by decreasing lactate levels from the start (mean [SD], 7.2 [3.2] mmol/L) to the end of perfusion (mean [SD], 1.21 [1.0] mmol/L) (to convert lactate levels to milligrams per deciliter, divide by 0.111) (eTable 1 in Supplement 2). The use of the OCS Liver significantly reduced the mean (SD) cold ischemia time of the donor livers to 175.4 (43.5) minutes compared with 338.8 (91.5) minutes for ICS (*P* < .001), despite the OCS Liver having significantly longer total cross-clamp (out of body) time (mean [SD], 454.9 [133.9] vs 338.8 [91.5] minutes) (*P* < .001). Overall, 155 DBD and DCD donor livers were assessed using the OCS Liver. Of those, 152 donor livers (98%) were successfully transplanted and analyzed in the PROTECT Trial. The 3 donor livers that failed OCS Liver assessment were from DCDs; 2 livers were rejected owing to lactate levels that continued to increase and reached higher than 10 mmol/L despite target flow parameters (Figure 1B), and 1 liver was rejected based on a pathologic finding of bridging fibrosis after recovery. We analyzed the effect of preservation modality on donor liver use for transplant from DBD and DCD donors. In this analysis, there was no difference in the percentage of DBD donor livers used between the OCS Liver or ICS group (124 of 154 [80%] in the OCS Liver group vs 133 of 168 [79%] in the ICS group). However, there was a significantly higher rate of DCD donor livers used for transplant associated with the OCS Liver compared with ICS (28 of 55 [51%] vs 13 of 51 [26%]; *P* = .007) (eFigure 3 in Supplement 2). The OCS Liver was readily integrated into 20 trial centers in which all teams were trained to perform all OCS Liver instrumentation, management, and assessment of donor livers during the entire preservation period in the trial. During the PROTECT trial, there were 3 minor device issues reported by trial centers (3 of 155 devices; 2%). Two issues were related to small plastic components in the perfusion module that did not interfere with the OCS Liver preservation or with the management of the donor livers on the system. One of the issues occurred well before the donor liver was retrieved prior to OCS Liver instrumentation. All 3 livers were transplanted successfully in the PROTECT Trial and their outcomes were analyzed in the results.

### Primary Effectiveness End Point

**Figure 3.**
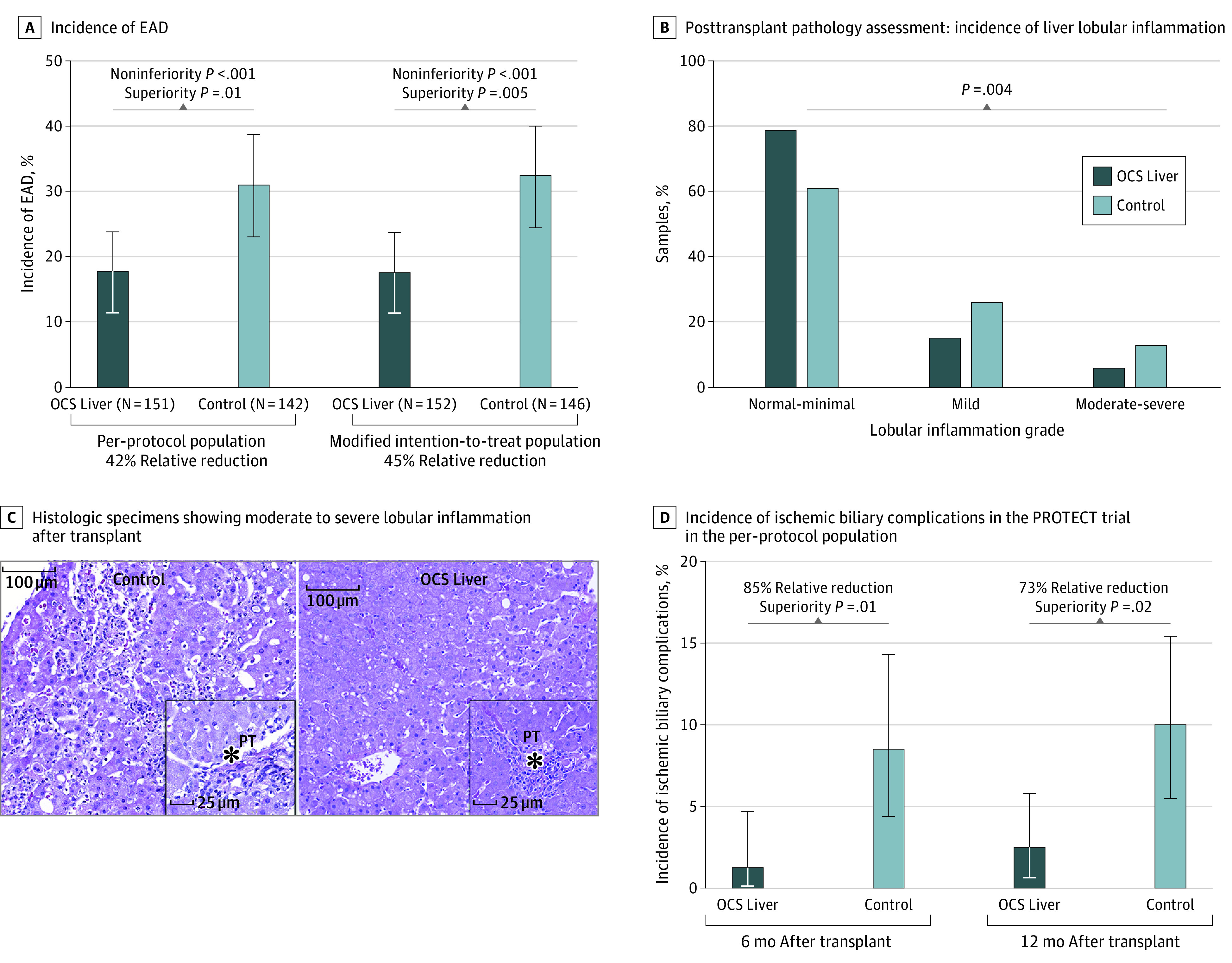
PROTECT Trial Primary Effectiveness End Point (Incidence of Early Allograft Dysfunction [EAD]), Posttransplant Pathology Assessment (Incidence of Liver Lobular Inflammation), Posttransplant Histology Representative Specimens of Moderate to Severe Lobular Inflammation, and PROTECT Trial Incidence of Ischemic Biliary Complications Within 12 Months After Transplant A, Clopper-Pearson exact CI for a binomial percentage, with 95% 1-sided upper confidence bound based on the Farrington-Manning score statistic. The noninferiority *P* values are based on the 1-sided Farrington-Manning score statistic, testing the null hypothesis that the true Organ Care System (OCS) Liver proportion is greater than or equal to the true ischemic cold storage (ICS) proportion δ = 0.075 vs the alternative hypothesis that it is less than the true ICS proportion plus 0.075. The superiority *P* values are from a 2-sided Fisher exact test, testing the null hypothesis that the true difference in proportions equals 0 vs the alternative hypothesis that it does not equal 0. This analysis was conducted only if the null hypothesis of inferiority was rejected. Error bars represent 95% CIs. B, The *P* value was determined using the χ^2^ test. C, Histologic specimens showing examples of severe lobular inflammation in a control ICS (left) liver after reperfusion, with inset showing minimal portal inflammation, and an OCS Liver–treated liver (right) showing absence of lobular inflammation and minimal portal inflammation (inset). The asterisk indicates the location of the portal tract (PT). D, Ischemic biliary complications, defined as nonanastomotic ischemic strictures or bile leaks, confirmed with an endoscopic retrograde cholangiopancreatography or magnetic resonance cholangiopancreatography radiologic examination.

**Figure 4.**
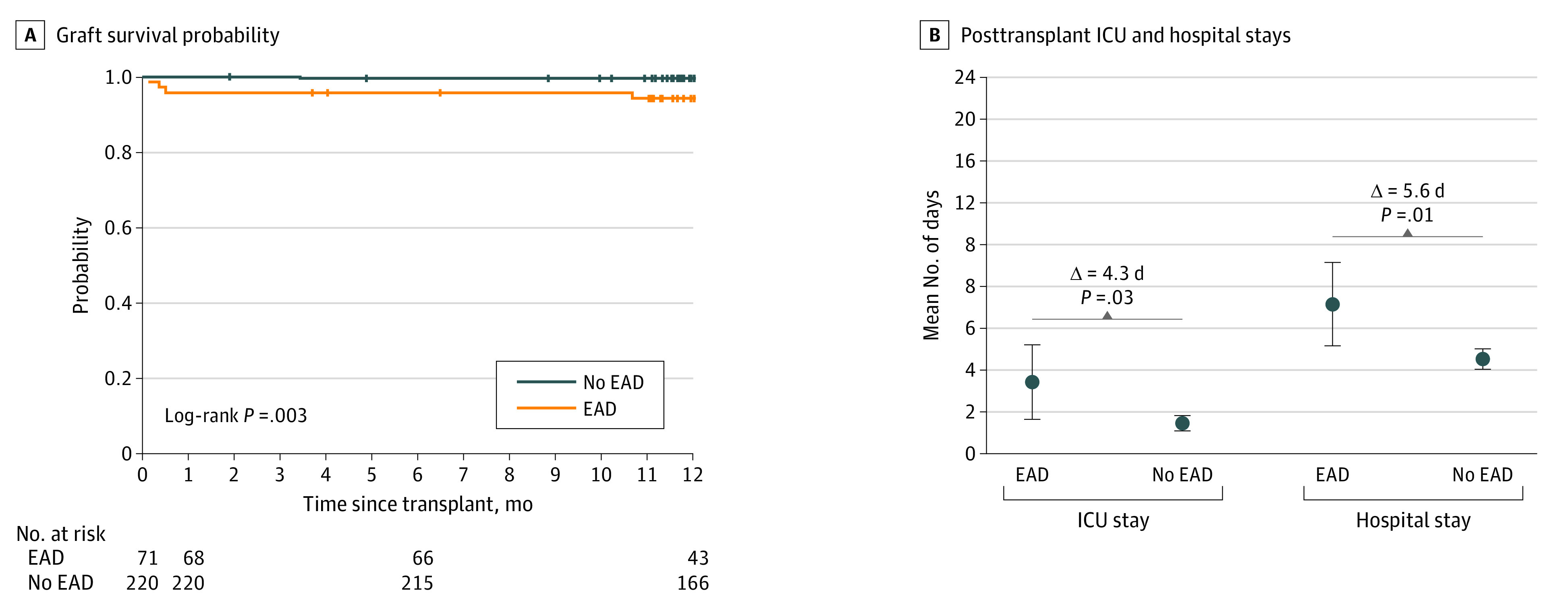
Effect of Early Allograft Dysfunction (EAD) in the Per-Protocol Population on Graft Survival Probability and on Posttransplant Intensive Care Unit (ICU) Stay and Overall Hospital Stay Error bars represent 95% CI.

The PROTECT trial met its primary effectiveness end point by demonstrating statistical noninferiority and superiority of outcomes of the OCS Liver group compared with the ICS group in both the per-protocol population and the modified intention-to-treat analysis population. Specifically, the use of the OCS Liver was associated with a significant decrease in the incidence of EAD compared with ICS in the primary analysis per-protocol population (27 of 151 [18%] vs 44 of 142 [31%]; *P* = .01). A similar effect was seen in the modified intention-to-treat population (Figure 3A). This significant reduction of EAD in the OCS group was further validated mechanistically by histopathologic assessment of liver graft biopsies after reperfusion. This assessment revealed that the OCS Liver was associated with significantly less lobular inflammation (for moderate to severe, 9 of 150 [6%] vs 18 of 141 [13%]; *P* = .004), a marker of ischemia-reperfusion injury^17,18,19^ (Figure 3B and C). By contrast, portal inflammation, a histologic marker not associated with ischemia-reperfusion injury, was similar between groups (moderate to severe, 2 of 150 [1.3%] vs 1 of 141 [0.7%]; *P* = .39). We examined the clinical effect of reducing EAD by stratifying all trial recipients outcomes by presence or absence of EAD. We found that EAD was associated with significantly higher risk of graft failure compared with no EAD (log-rank test *P* = .003) (Figure 4A), significantly longer ICU stay (mean [SD], 7.7 [16.9] vs 3.4 [4.8] days; *P* = .04) and significantly longer overall hospital stay (mean [SD], 15.7 [19.0] vs 10.1 [7.9] days; *P* = .02) (Figure 4A and B).

### Secondary and Other Critical Clinical End Points

The OCS Liver permitted continuous monitoring and assessment of lactate levels, bile production, and hepatic artery and portal vein pressures and flows throughout preservation (eTable 1 in Supplement 2). Patient survival at day 30 after transplant was 99.3% (150 of 151) for the OCS Liver group vs 99.3% (141 of 142) for the ICS group (noninferiority *P* < .001), and at initial hospital discharge, patient survival was 98.7% (149 of 151) for the OCS Liver group vs 98.6% (140 of 142) for the ICS group (noninferiority *P* < .001). The OCS Liver was associated with a significant decrease in ischemic biliary complications 6 months (2 of 151 [1.3%] vs 12 of 142 [8.5%]; *P* = .02) and 12 months (4 of 151 [2.6%] vs 14 of 142 [9.9%]; *P* = .02) after transplant (Figure 3D). Reperfusion syndrome was more severe in the ICS group compared with the OCS Liver group based on significantly higher recipient lactate levels at approximately 120 minutes after reperfusion (mean [SD], 3.64 [2.22] vs 4.33 [2.98] mmol/L; *P* = .046).^20^ There was no difference in the incidence of anastomotic nonischemic biliary complications (eTable 2 in Supplement 2). Overall 12-month patient survival was 94.0% (142 of 151) for the OCS Liver group and 93.7% (133 of 142) for the ICS group (eFigure 4A and B in Supplement 2).

### Safety End Point

The PROTECT trial met its safety end point showing that the mean (SD) number of LGRSAEs within the initial 30 days after transplant for the OCS Liver was noninferior to ICS (mean [SD], 0.046 [0.210] per patient in the OCS Liver group vs 0.075 [0.265] per patient in the ICS group; noninferiority *P* < .001). The type of LGRSAEs observed within the initial 30 days after transplant revealed an apparent decrease for the OCS Liver vs ICS in both IBCs (0 vs 2 events in 2 patients) and vascular complications (8 events in 7 patients vs 11 events in 9 patients) (eTable 3 in Supplement 2).

## Discussion

Until the recent development of perfusion strategies,^12,13,14,15,16,21,22,23,24,25,26,27,28^ solid organ preservation had been restricted to ICS, with the concern of resultant ischemia-reperfusion injury^5,8^ contributing to underuse of donor organs for transplant. The results of this randomized clinical trial showed that portable normothermic machine perfusion, the OCS Liver, was superior to ICS, conferring a lower incidence of EAD and IBCs in recipients of liver allografts.

Some clinicians have championed using in situ normothermic regional perfusion in the donor body prior to DCD liver allograft retrieval.^27,29^ This approach has not been broadly adopted owing to the complexity and impracticality of routine implementation during multiorgan recovery and because a period of cold storage generally follows normothermic regional perfusion. Nasralla et al^16^ reported the first European randomized clinical trial of normothermic machine perfusion in 220 liver transplants and showed early significant reduction in peak enzymes in the normothermic machine perfusion group. However, protection from IBC was not observed, and there was no reported mechanistic explanation for the peak enzyme differences. A recent nonrandomized study used end-ischemic normothermic machine perfusion for donor livers that would have otherwise been discarded.^30^ Donor livers in that trial had a median of more than 8 hours of ICS prior to commencing normothermic machine perfusion, perhaps explaining the disappointing results that included a rate of IBC requiring retransplant of 30% among recipients of DCD grafts. These findings further highlight the importance of portability to a normothermic liver perfusion system, enabling initiation of perfusion at the donor hospital to maximally reduce ICS.

In contrast to these efforts, the hypothermic oxygenated perfusion at the end of a cold ischemic period (HOPE) protocol recently showed impressive reduction in EAD and prevention of IBC in DCD liver transplant.^31^ Thus, HOPE and OCS approaches may have complementary applications depending on the clinical situation. However, cold perfusion is suboptimal to assess the transplantability of steatotic livers and may therefore not be relevant in the assessment of organs currently being discarded after recovery or not deemed worthy of recovery.^32^

The PROTECT trial was designed to overcome the significant limitations of ICS and to evaluate the clinical effect of reducing ischemic injury and providing functional assessment of liver allografts using a portable normothermic perfusion system. The use of the OCS Liver resulted in a significant reduction in ischemic injury associated with ICS and led to superior short-term and midterm clinical outcomes, with significant reduction of EAD and IBC through 12 months’ follow-up. The PROTECT trial provided clear mechanistic corroboration of the marked reduction of EAD observed in the trial based on the significant decrease in the histologic marker of ischemia-reperfusion injury, lobular inflammation, observed on histologic specimens recovered following reperfusion of the graft in the recipient, and significant mitigation of reperfusion syndrome in the recipient after transplant in the OCS group. The clinical importance of reducing EAD was reflected in significantly shorter ICU and hospital stays, and significant improvement in posttransplant graft survival. The PROTECT trial met its safety end point and reported low rates of LGRSAEs.

The PROTECT trial targeted DCD donors and DBD donors having risk factors that included older age, moderate level of steatosis, or anticipated long cross-clamp time, that portend EAD, primary nonfunction, or IBC. Use of the OCS Liver resulted in significantly increased DCD donor use as compared with ICS. These data suggest that the OCS Liver provided additional opportunity for ex vivo clinical optimization and assessment of the DCD liver graft, resulting in a higher proportion of DCD livers being successfully transplanted with the use of the OCS Liver compared with ICS. These results confirm the inherent clinical benefits of normothermic machine perfusion to provide an additional clinical quality assessment of liver allografts. Increasing use of DCD and questionable DBD liver allografts by demonstrating their viability and suitability for transplantation will likely be the greatest clinical benefit of ex vivo perfusion in liver transplant. The portability of the OCS Liver will likely gain increasing importance as organ retrieval teams travel further to recover organs with recently adopted allocation rules that mandate broader sharing of donor livers in the US.^33^

### Limitations

Complete blinding of the clinical teams to the preservation modality was impossible given the complex logistics involved in the application of machine perfusion at a remote site. Therefore, the randomization step was designed to occur only after the clinical decision of accepting the donor offer had been made based on clinical facts about the donor and without any knowledge of the preservation modality. If for any reason, the donor liver was rejected for transplant on physical examination during organ retrieval, the protocol prespecified that the potential recipient be rerandomized after a second donor liver was offered and clinically accepted. This process was established to ensure that clinical decision-making was the primary driver for accepting donor liver allografts and was not influenced by preservation modality. The balanced donor and recipient characteristics and risk factors as well as the equivalent baseline donor liver pathology assessment validate the robustness of this randomization process.

## Conclusions

The PROTECT trial showed superior short-term and mid-term clinical outcomes and higher numbers of donor livers used for transplant. The advent of portable, extracorporeal, donor liver machine perfusion offers for the first time a convenient and effective approach to both assess and enhance donor liver function, thereby improving transplant safety, expanding the liver donor pool, and reducing waiting list mortality.

## Notes

Supplement 1. Trial Protocol

Supplement 2. eTable 1. OCS Liver Perfusion Parameters, Bile Production and Perfusion Chemistry eTable 2. Bile Duct-Related Complications Through 12 Months eTable 3. Number of Liver Graft–Related Serious Adverse Events (LGRSAEs) During the First 30 Days After Transplantation in the PROTECT Trial eFigure 1. Detailed Description of the PROTECT Randomization Procedure eFigure 2. Animation of OCS Liver Unique Flow Adjustment for Delivery of Hepatic Artery and Portal Vein Flow Using a Single Pulsatile Pump eFigure 3. Utilization of DCD and DBD Donors in PROTECT eFigure 4. PROTECT Trial Patient Survival (A) and Graft Survival (B) Through 12 Months (PP Population)

Supplement 3. Data Sharing Statement
