# Supplementary material for: Impact of Portable Normothermic Blood-Based Machine Perfusion on Outcomes of Liver Transplant: The OCS Liver PROTECT Randomized Clinical Trial
Source: JAMA Surg. 2022 Jan 5;157(3):189–98. doi: 10.1001/jamasurg.2021.6781 (PMC8733869; doi:10.1001/jamasurg.2021.6781)
Supplement: Supplement 1. — Trial Protocol [file jamasurg-e216781-s001.pdf]

International Randomized Trial to Evaluate The Effectiveness of The Portable Organ Care System (OCS™) Liver For Preserving and Assessing Donor Livers for Transplantation (OCS Liver PROTECT Trial)

OCS™ Liver PROTECT Trial

Protocol Number OCS-LVR-072014

July 2014

Sponsor: TransMedics, Inc.
200 Minuteman Road, Suite 302
Andover, MA 01810

Sponsor Primary Contact: Waleed Hassanein, MD
President & CEO
Phone: (978) 552-0901
Fax: (781) 998-0354
Email: whassanein@transmedics.com

Sponsor Secondary Contact: Tamer Khayal, MD
Chief Medical Officer
Phone: (978) 552-0902
Fax: (978) 552-0978
Email: tkhayal@transmedics.com

<u>CONFIDENTIAL – PROPRIETARY INFORMATION</u>

OCS™ Liver PROTECT Trial Synopsis

**Protocol Title**	International Randomized Trial to Evaluate The Effectiveness of The Portable Organ Care System (OCS™) Liver For Preserving and Assessing Donor Livers for Transplantation (OCS™ Liver PROTECT Trial)
**Objectives**	To evaluate the effectiveness of the OCS™ Liver to preserve and assess donor livers intended for transplantation
**Trial Design**	A prospective, pivotal, international randomized trial
**Trial Size**	A maximum of 20 participating sites worldwide with 300 transplanted liver recipients
**Screening and Treatment**	Donor livers will be screened for trial eligibility. Eligible donor livers will be preserved either using the OCS™ Liver system (OCS group) or cold flush and storage using cold preservation solution (SOC group)<br><br>Primary Liver transplant candidates will be screened for trial eligibility. Every eligible candidate will be asked to participate. Eligible liver transplant candidates will be randomized to receive either a donor liver preserved either on OCS™ Liver (OCS) or using cold preservation solution (SOC)
**Donor Liver Eligibility Criteria**	**Inclusion**<br>Donor livers suitable for preservation with either OCS or SOC and meets at least one of the following:<br>• Donor age ≥40 years old<br>• Expected cross clamp time ≥4 hours<br>• Donor after circulatory death (DCD) with age ≤ 55 years old<br>• Steatotic liver ≤ 40% macrosteatosis at time of retrieval (based on clinical diagnosis or histology at time of liver retrieval)<br><br>**Exclusion**<br>• Liver in which investigator is unwilling to randomize to either arm<br>• Living donors<br>• Liver intended for split transplants<br>• Positive serology (HIV, Hepatitis B & C)<br>• Presence of moderate or severe traumatic liver injury
**Recipient Eligibility Criteria**	**Inclusion**<br>• Registered male or female primary Liver transplant candidate<br>• Age ≥18 years old<br>• Signed: 1) written informed consent document and 2) authorization to use and disclose protected health information

	**Exclusion**<br>• Acute, fulminant liver failure<br>• Prior solid organ or bone marrow transplant<br>• Chronic use of hemodialysis or diagnosis of chronic renal failure, defined as chronic serum creatinine of >3 mg/dl for >2 weeks and/or requiring hemodialysis<br>• Multi-organ transplant
**Primary Endpoint**	Primary liver graft dysfunction defined as presentation of one or more of the following criteria:<br>• AST level > 2000 IU/ml within the first 7 postoperative days;<br>• Bilirubin ≥ 10 mg/dl on postoperative day 7;<br>• INR ≥ 1.6 on postoperative day 7; or<br>• Primary non-functioning graft
**Secondary Endpoints**	• Patient survival at day-30 post transplantation<br>• Graft survival at day-30 post transplant<br>• Need for Renal replacement therapy, defined as requiring >2 dialysis treatments in the first 10 days post liver transplantation
**Other Endpoints**	• Length of initial post-transplant ICU stay<br>• Length of initial post-transplant hospital stay<br>• Evidence of biliary strictures diagnosed at 6, 12 or 24 months
**Safety Endpoints**	Incidence of liver graft related Serious Adverse Events (SAEs) in the first 30 days post liver transplantation, defined as:<br>• Primary non-function (defined as irreversible graft dysfunction requiring emergency liver re-transplantation or death with the first 10 days, in the absence of immunologic or technical causes);<br>• Biopsy proven acute rejection;<br>• Biliary complications (biliary strictures, anastomotic and non-anastomotic bile duct leaks);<br>• Vascular complications (non-surgical bleeding, hepatic artery stenosis, hepatic artery thrombosis and portal vein thrombosis);<br>• Liver graft related infections (liver abscess, cholangitis, etc.)
**Follow-up**	All patients will be followed for up to 2 years post-transplant.
**Trial Sponsor**	TransMedics, Inc.<br>200 Minuteman Road, Suite 302<br>Andover, MA, USA 01810
