# Supplementary material for: Impact of Portable Normothermic Blood-Based Machine Perfusion on Outcomes of Liver Transplant: The OCS Liver PROTECT Randomized Clinical Trial
Source: JAMA Surg. 2022 Jan 5;157(3):189–98. doi: 10.1001/jamasurg.2021.6781 (PMC8733869; doi:10.1001/jamasurg.2021.6781)
Supplement: Supplement 2. — eTable 1. OCS Liver Perfusion Parameters, Bile Production and Perfusion Chemistry eTable 2. Bile Duct-Related Complications Through 12 Months eTable 3. Number of Liver Graft–Related Serious Adverse Events (LGRSAEs) During the First 30 Days After Transplantation in the PROTECT Trial eFigure 1. Detailed Description of the PROTECT Randomization Procedure eFigure 2. Animation of OCS Liver Unique Flow Adjustment for Delivery of Hepatic Artery and Portal Vein Flow Using a Single Pulsatile Pump eFigure 3. Utilization of DCD and DBD Donors in PROTECT eFigure 4. PROTECT Trial Patient Survival (A) and Graft Survival (B) Through 12 Months (PP Population) [file jamasurg-e216781-s002.pdf]

Supplementary Online Content

Markmann JF, Abouljoud MS, Ghobrial RM, et al. Impact of portable normothermic blood-based machine perfusion on outcomes of liver transplant: the OCS Liver PROTECT randomized clinical trial. *JAMA Surg*. Published online January 5, 2022. doi:10.1001/jamasurg.2021.6781

eTable 1. OCS Liver Perfusion Parameters, Bile Production and Perfusion Chemistry
eTable 2. Bile Duct-Related Complications Through 12 Months
eTable 3. Number of Liver Graft–Related Serious Adverse Events (LGRSAEs) During the First 30 Days After Transplantation in the PROTECT Trial
eFigure 1. Detailed Description of the PROTECT Randomization Procedure
eFigure 2. Animation of OCS Liver Unique Flow Adjustment for Delivery of Hepatic Artery and Portal Vein Flow Using a Single Pulsatile Pump
eFigure 3. Utilization of DCD and DBD Donors in PROTECT
eFigure 4. PROTECT Trial Patient Survival (A) and Graft Survival (B) Through 12 Months (PP Population)

This supplementary material has been provided by the authors to give readers additional information about their work.

© 2022 Markmann JF et al. *JAMA Surgery*.

eTABLE 1. OCS Liver Perfusion Parameters, Bile Production and Perfusate Chemistry

OCS Perfusion Parameters & Perfusate Chemistry	OCS (N=152)
OCS Liver Perfusion Time (mins) – mean ± SD	276.6 ± 117.4
Hepatic Artery Pressure (mmHg) – mean ± SD	70.6 ± 16.2
Hepatic Artery Flow (L/min) – mean ± SD	0.7 ± 0.2
Portal Vein Pressure (mmHg) – mean ± SD	5.4 ± 2.3
Portal Vein Flow (L/min) – mean ± SD	1.3 ± 0.1
Total Bile Production (ml) – mean ± SD	28.3 ± 15.9
pH – mean ± SD	7.43 ± 0.1
PaO_2 (mmHg) – mean ± SD	420.2 ± 80.7
PCO2 (mmHg) – mean ± SD	41.5 ± 14.6
HCO3 (mEq/L) – mean ± SD	28.6 ± 10.3
OCS Liver Perfusate Lactate Trend:	
Starting Lactate (mmol/L) – mean ± SD	7.2 ± 3.2
Ending Lactate (mmol/L) – mean ± SD	1.2 ± 1.0

© 2022 Markmann JF et al. *JAMA Surgery*.

eTABLE 2. Biliary Duct-Related Complications Through 12 Months

	OCS (N= 153)	Control (N= 146)	
Biliary complications	Number of Patients (%)	Number of Patients (%)	P value
**Ischemic biliary complications**	**4 (2.6%)**	**14 (9.6%)**	**P=0.02**
**Anastomotic complications**	**17 (11.1%)**	**17 (11.6%)**	**P=1.0**

P-values from a two-sided Fisher's Exact Test

© 2022 Markmann JF et al. *JAMA Surgery*.

eTABLE 3. Number of Liver Graft–Related Serious Adverse Events (LGRSAEs) During the First 30 Days After Transplantation in the PROTECT Trial

	OCS (N= 153)		Control (N= 146)	
	Number of Patients (%)	Number of Events (% of total events)	Number of Patients (%)	Number of Events (% of total events)
Patients with at least one LGRSAE within 30 days post-transplant	7 (5%)	8	11 (8%)	13
Non-functioning graft	0	0	0	0
Ischemic biliary complications	0	0	2 (1%)	2 (15%)
Vascular complications	7 (5%)	8 (100%)	9 (6%)	11 (85%)
Liver allograft infections	0	0	0	0

© 2022 Markmann JF et al. *JAMA Surgery*.

eFigure 1. Detailed Description of the PROTECT Randomization Protocol

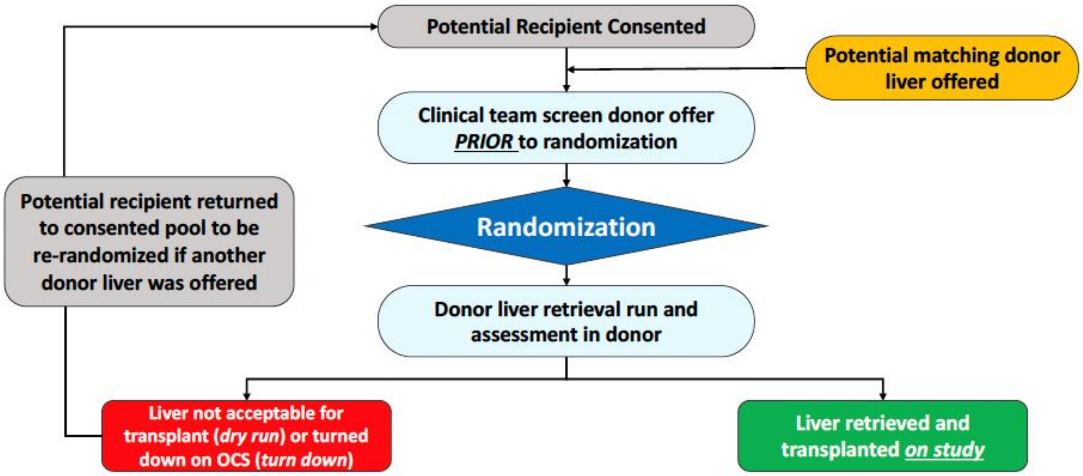

© 2022 Markmann JF et al. *JAMA Surgery*.

eFigure 2. Animation of OCS Liver Unique Flow Adjustment for Delivery of Hepatic Artery and Portal Vein Flow Using a Single Pulsatile Pump

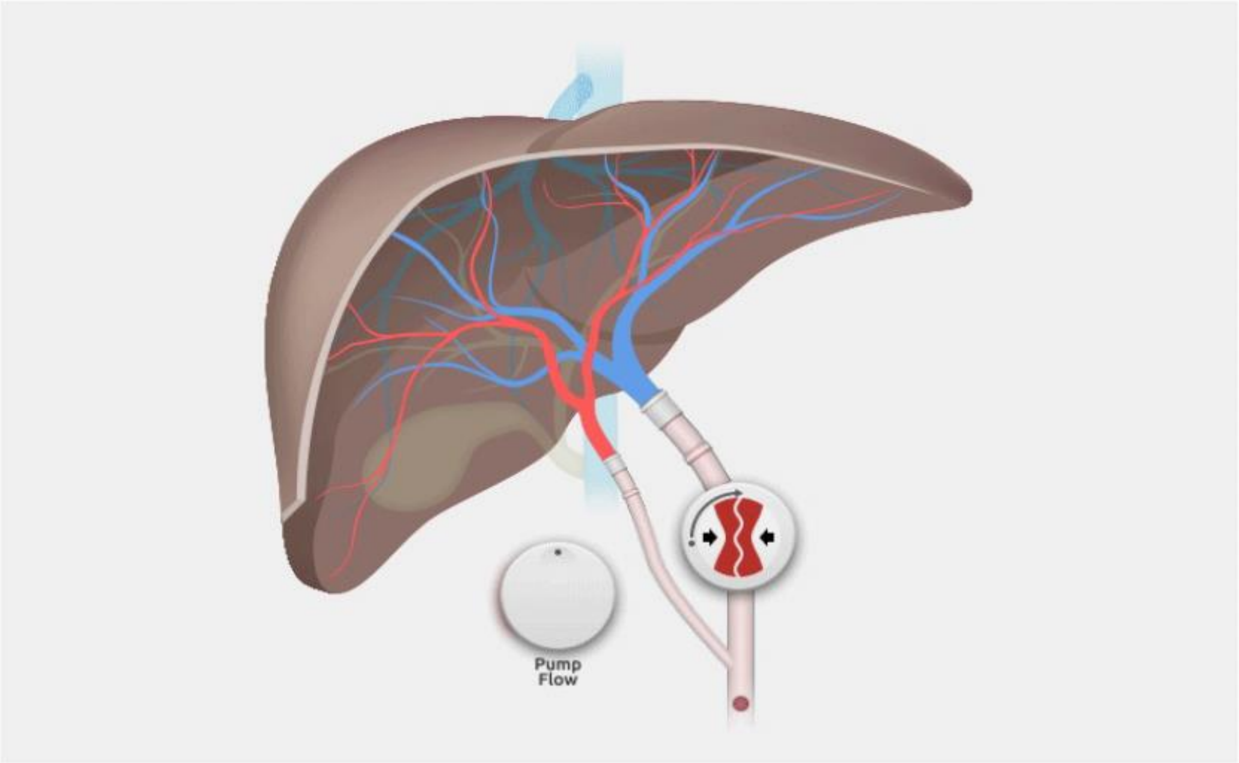

© 2022 Markmann JF et al. *JAMA Surgery*.

eFigure 3. Utilization of DCD and DBD Donors in PROTECT

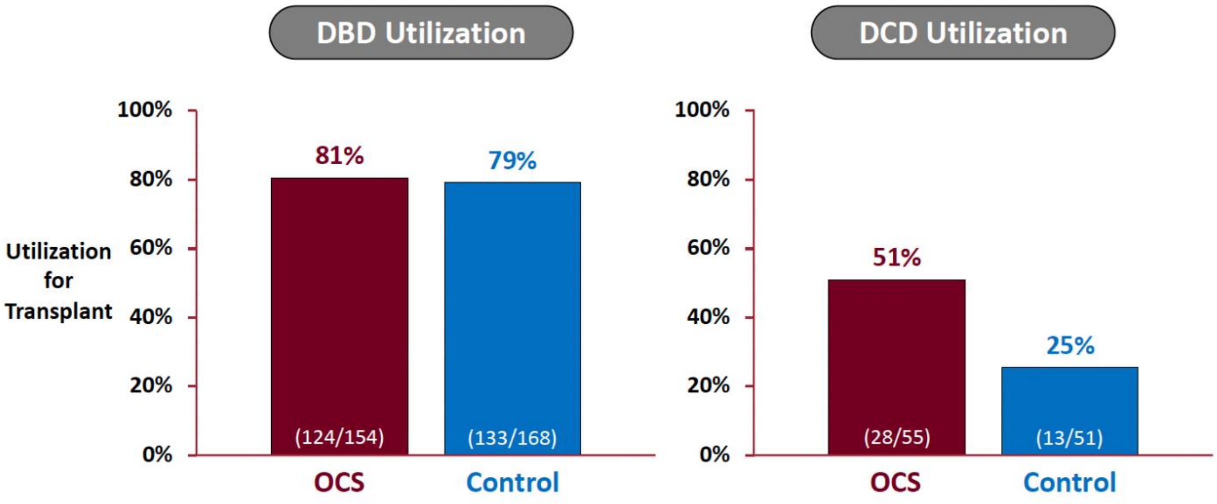

© 2022 Markmann JF et al. *JAMA Surgery*.

eFigure 4. PROTECT Trial Patient Survival (A) and Graft Survival (B) Through 12 Months (PP Population)

A. Patient Survival

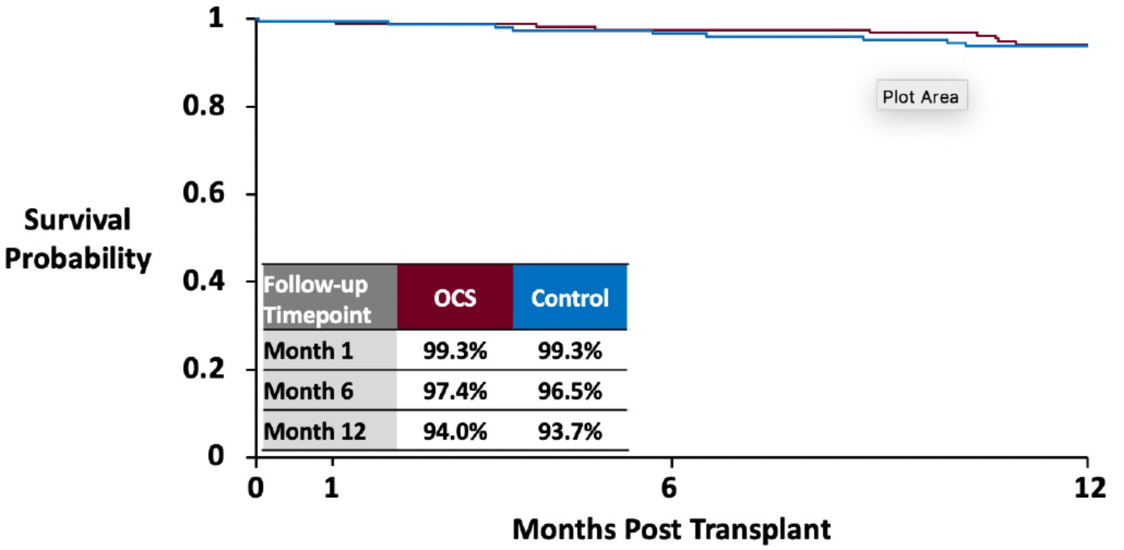

OCS	151	150	147	105
Control	142	141	137	106

B. Graft Survival

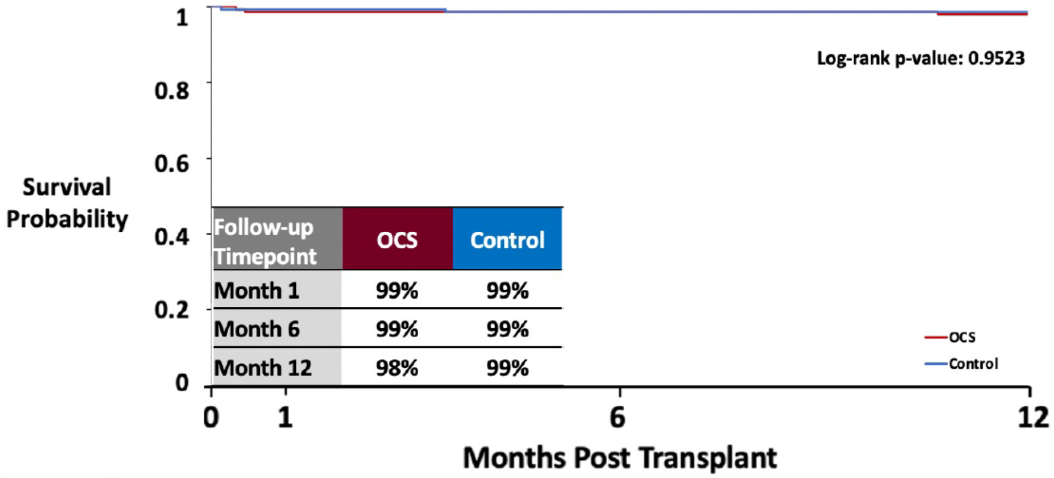

OCS	151	148	145	104
Control	142	140	136	105

© 2022 Markmann JF et al. *JAMA Surgery*.
