# Supplementary material for: Impact of Portable Normothermic Blood-Based Machine Perfusion on Outcomes of Liver Transplant: The OCS Liver PROTECT Randomized Clinical Trial
Source: JAMA Surg. 2022 Jan 5;157(3):189–98. doi: 10.1001/jamasurg.2021.6781 (PMC8733869; doi:10.1001/jamasurg.2021.6781)
Supplement: Supplement 3. — Data Sharing Statement [file jamasurg-e216781-s003.pdf]

Data Sharing Statement

Markmann. Impact of Portable Normothermic Blood-Based Machine Perfusion on Outcomes of Liver Transplant. *JAMA Surg*. Published January 05, 2022. doi:10.1001/jamasurg.2021.6781

Data
Data available: No

Additional Information
Explanation for why data not available: Data is still under consideration by FDA.
